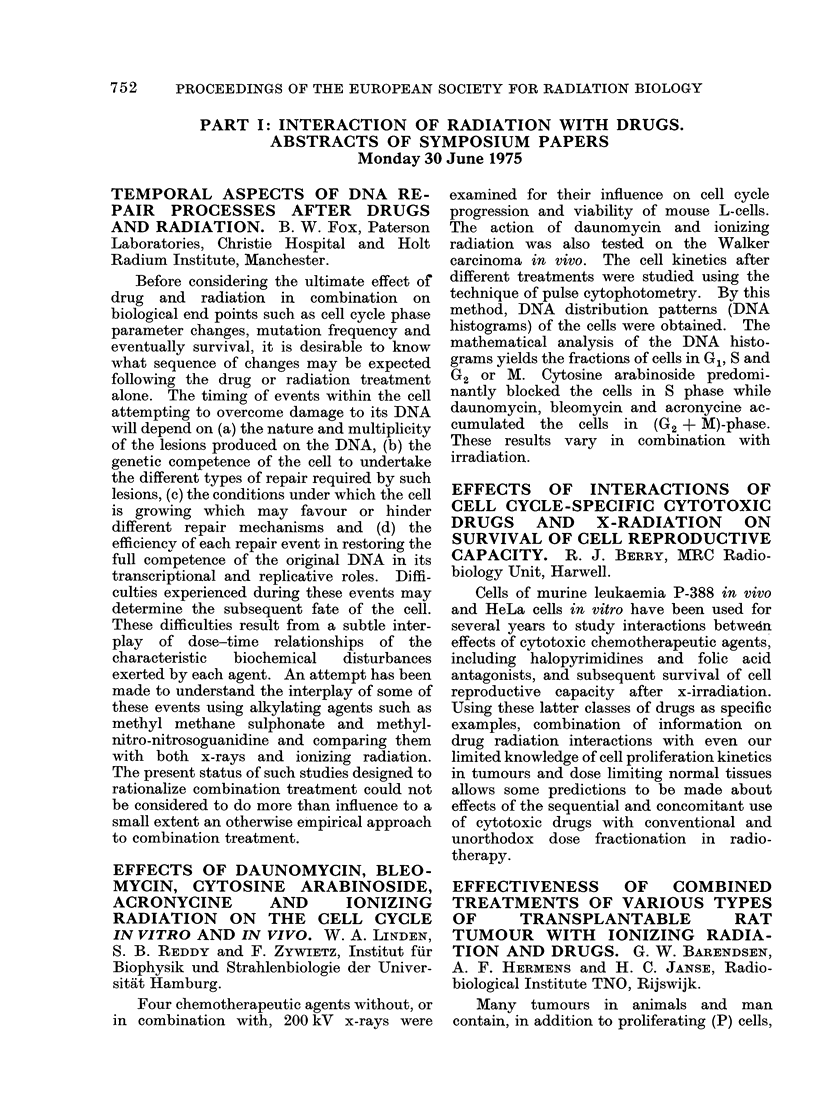# Proceedings: Effects of daunomycin, bleomycin, cytosine arabinoside, acronycine and ionizing radiation on the cell cycle in vitro and in vivo.

**DOI:** 10.1038/bjc.1975.293

**Published:** 1975-12

**Authors:** W. A. Linden, S. B. Reddy, F. Zywietz


**EFFECTS OF DAUNOMYCIN, BLEOMYCIN, CYTOSINE ARABINOSIDE, ACRONYCINE AND IONIZING RADIATION ON THE CELL CYCLE *IN VITRO* AND *IN VIVO*.** W. A. Linden, S. B. Reddy and F. Zywietz, Institut für Biophysik und Strahlenbiologie der Universität Hamburg.

Four chemotherapeutic agents without, or in combination with, 200 kV x-rays were examined for their influence on cell cycle progression and viability of mouse L-cells. The action of daunomycin and ionizing radiation was also tested on the Walker carcinoma *in vivo*. The cell kinetics after different treatments were studied using the technique of pulse cytophotometry. By this method, DNA distribution patterns (DNA histograms) of the cells were obtained. The mathematical analysis of the DNA histograms yields the fractions of cells in $G_1$, S and $G_2$ or M. Cytosine arabinoside predominantly blocked the cells in S phase while daunomycin, bleomycin and acronycine accumulated the cells in ($G_2 + M$)-phase. These results vary in combination with irradiation.